# The Effect of Yogurt Consumption on Body Fat Percentage in School-Age Children: A Quantile Regression Analysis

**DOI:** 10.3390/nu18050780

**Published:** 2026-02-27

**Authors:** Tingting Gao, Wei Cao, Titi Yang, Peipei Xu, Juan Xu, Qian Gan, Hongliang Wang, Hui Pan, Yingying Zhao, Fengshuang Wang, Wenhua Zhao, Zhenyu Yang, Qian Zhang

**Affiliations:** 1 Beijing Shunyi District Center for Disease Control and Prevention, Beijing 101300, China; gtt0203@126.com (T.G.);; 2 NHC Key Laboratory of Trace Element Nutrition, National Institute for Nutrition and Health, Chinese Center for Disease Control and Prevention, Beijing 100050, China

**Keywords:** children, yogurt, consumption, body fat percentage

## Abstract

**Objectives**: The objective of this study was to investigate the association between yogurt consumption and body fat percentage among Chinese children and to analyze the potential influence of factors such as sex, pubertal stage, and physical activity level on this association. **Methods**: This study conducted a nationwide survey using multi-stage stratified random sampling, including 48,305 children aged 6–17 years. Body fat percentage (BFP) was measured using bioelectrical impedance analysis (BIA). Daily yogurt consumption was adjusted using the density method (daily yogurt consumption = daily yogurt consumption × 1000/total energy consumption). The association between yogurt consumption and body fat percentage was analyzed using quantile regression, with covariates adjusted for participants’ age, pubertal development stage, geographical region, total daily energy consumption, physical activity duration, annual household income, parental educational attainment, and consumption of other food categories. **Results**: Boys aged 6–10 years, 11–14 years, and 15–17 years had median daily yogurt consumptions of 28.6 g, 28.6 g and 21.4 g per day, with BFP values of 19.6, 19.5 and 17.5. Girls in the same age groups showed consumption of 28.6 g, 29.6 g and 28.6 g, with BFP values of 20.3, 26.4 and 31. The quantile regression results for boys showed that daily consumption of yogurt was significantly correlated with their BFP at the 0.25, 0.50, 0.75, 0.85 and 0.95 quartiles, with regression coefficients of −0.207, −0.300, −0.688, −0.570, and −0.465 after adjusting for potential confounders. For girls, there was a significant correlation in the 0.75 and 0.85 quartiles, with regression coefficients of −0.290 and −0.582, after adjusting for potential confounders. **Conclusions**: A significant inverse association was observed between yogurt consumption and body fat percentage, with notable differences between boys and girls. Further intervention studies are warranted to evaluate the long-term effects of incorporating yogurt into the diets of Chinese children and adolescents on body fat percentage and obesity risk.

## 1. Introduction

In recent years, the rising global prevalence of childhood obesity has emerged as a critical public health issue, posing a significant threat to the well-being of children worldwide [1]. In 2016, over 340 million children and adolescents aged 5–19 years worldwide were affected by overweight and obesity, a number that had exceeded 390 million by 2022 [2]. In China, the prevalence of overweight and obesity among children is also on the rise [3]. Studies indicate that by 2020, over 19.0% of children and adolescents aged 5–19 years in China were affected by overweight and obesity [4], a figure significantly higher than the 10.2% reported in 2012. Abnormal elevation in body fat percentage is a core pathological feature of obesity [5]. It not only affects the quality of growth and development during childhood but also increases the risk of chronic diseases in adulthood, such as type 2 diabetes and cardiovascular diseases [6,7]. Childhood is a critical window for growth and development, during which body fat metabolism is highly malleable [8,9,10]. Therefore, identifying the influencing factors of children’s body fat percentage is of significant practical importance for the prevention and control of obesity. Dairy products, rich in high-quality protein, calcium, and bioactive compounds (such as milk-derived opioid peptides, e.g., β-casomorphin-7, and casein phosphopeptides), have become a research focus for their association with children’s body fat metabolism [11,12]. As an important category of dairy products, yogurt offers more advantages compared to fresh milk [13,14]. First, during the fermentation process, approximately 20–30% of lactose is degraded, which renders yogurt more easily digestible and absorbable for children [15]. Secondly, yogurt is fermented from cow’s milk as the substrate by inoculating probiotic starter strains such as Streptococcus thermophilus and Lactobacillus bulgaricus [16]. During fermentation, these probiotics metabolize lactose to produce acid and proliferate in large quantities, forming the core probiotic flora; the additional strains added in fortified yogurt, like Lactobacillus rhamnosus, also proliferate synchronously, enriching the variety and viable count of probiotics in yogurt. These fermentation-derived probiotics serve as the core material basis for yogurt to exert its physiological effects and may indirectly participate in body fat regulation by modulating the structure of the gut microbiota, thereby influencing the body’s energy metabolism and appetite regulation [17]. Additionally, yogurt also serves as a source of high-quality protein. Its relatively high protein content can delay gastric emptying and enhance satiety, potentially helping to reduce total energy consumption in children and thus exerting a regulatory effect on body fat percentage [18,19]. Some observational studies in adults have indicated that yogurt consumption is associated with a reduction in body fat percentage [20]. For instance, a study involving 720 Australian adults classified as having overweight or obesity reported that the participants had an average daily yogurt consumption of 53 ± 66 g. Through linear regression analysis, it was found that yogurt consumption was inversely associated with body fat percentage (β = −0.014, *p* < 0.05), after controlling for variables such as age, gender, and total energy consumption [21].

Nevertheless, other studies have pointed out that commercially available yogurt often contains large amounts of added sucrose, which might offset the nutritional advantages of yogurt itself, even increasing the risk of body fat accumulation. A cross-sectional study from Poland in 1172 preschool children found that the frequency of fruit yogurt consumption was positively correlated with body fat percentage (BFP) in the total sample and in girls, with a correlation coefficient of 0.10–0.39. Moreover, their frequency of natural yogurt consumption was positively correlated with body fat (r = 0.081, r = 0.098, *p* < 0.05) [22]. Therefore, there remains significant divergence in current research conclusions regarding the relationship between yogurt consumption and body fat percentage.

Existing studies have several limitations, including small sample sizes, a primary focus on Western populations, and a lack of large-scale targeted studies on Chinese children. Chinese children exhibit significant differences in dietary patterns and gut microbiota characteristics compared to their Western counterparts, making direct application of international research conclusions potentially biased. Additionally, many studies fail to adequately account for confounding factors such as overall dietary structure (e.g., total energy consumption and dietary fiber consumption) and physical activity levels, which limits the applicability and reliability of the findings.

Given this context, the present study aims to analyze the association between yogurt consumption and body fat percentage in Chinese school-aged children through a cross-sectional survey and offer new strategies for the early prevention and management of childhood obesity.

## 2. Materials and Methods

### 2.1. Study Population

This study employed a multi-stage stratified random sampling method to survey children aged 6–17 years from schools (primary, junior high, and senior high schools) across 28 districts/counties in 14 provinces of China. At each survey site, 196 participants were investigated for each one-year age group, resulting in a total of 2352 individuals per site. Across all 28 survey sites nationwide, approximately 66,000 children aged 6–17 years were included in the survey. Inclusion criteria for the study participants were as follows: (1) locally born healthy children aged 6–17 years, and (2) children who have resided in the survey area for more than 6 months and have no plans to relocate within the next year. The exclusion criterion was children who had been diagnosed with any acute or chronic disease by a secondary or higher-level hospital. Ultimately, a total of 58,101 valid questionnaires were collected.

The study received ethical approval from the Institutional Review Board of the National Institute for Nutrition and Health, Chinese Center for Disease Control and Prevention. Prior to enrollment, written informed consent was provided by the legal guardians of all participating children.

### 2.2. Dietary Survey

The food frequency questionnaire (FFQ) was used to obtain children’s consumption of yogurt. The questionnaire uses face-to-face questions to ask study participants about their average daily consumption of various foods over the past month. The survey covered 72 food items across 12 categories, namely cereals and potatoes, soy products, vegetables, fruits, dairy products, livestock and poultry meat, aquatic products, eggs, nuts, beverages, water, and snacks. Consumption of dairy products by school-age children—including fresh milk, yogurt, milk powder, cheese, etc.—was further converted to fresh milk equivalents based on their protein content. Total daily energy consumption was determined using the Chinese standard food composition tables [23,24]. The daily dairy product consumption was adjusted using the density method, with the adjusted value calculated as follows: daily dairy product consumption (g/1000 kcal) = daily dairy product consumption × 1000/total energy consumption [25].

### 2.3. Body Fat Percentage

Children’s body composition was measured using bioelectrical impedance analysis (BIA). Bioelectrical impedance devices (InBody 770, Inbody Co., Ltd., Seoul, Republic of Korea) adopt the hand-to-foot bioelectrical impedance analysis (hand-to-foot BIA) technology. They analyze human body composition using the multi-loop method, with eight tactile electrodes for the hands and feet and six frequency bands (1, 5, 50, 250, 500, and 1000 kHz). These impedance values are then used to estimate multiple variables, including body fat percentage (BFP), fat mass (FM), fat-free mass (FFM), and other indicators [26]. To ensure measurement accuracy, subjects were required to be in a fasting state in the morning, empty their bladder and bowels, remove excess items such as mobile phones and keys, wear lightweight indoor clothing, and take off socks to keep their weight as close as possible to their bare weight. Additionally, participants were required to avoid strenuous exercise and bathing within two hours before the test and to skip body composition measurements during menstruation.

### 2.4. Covariates

The weekly duration of time spent on high-intensity physical activity (h/w) and sedentary behavior (h/w) was obtained through the Chinese Physical Activity Questionnaire for Children Aged 6–17 Years (CCPAQ) [27], established and validated by this project. The questionnaire effectively distinguishes between types and patterns of physical activity and reliably measures the time children spend on physical activity during the week (both school days and weekends).

Pubertal developmental status was classified using the Tanner five-stage method [26]. The Tanner staging system is a standardized framework that assesses pubertal development through five progressive stages: Stage I (prepubertal state), Stage II (initial development of secondary sexual characteristics), Stage III (accelerated growth phase), Stage IV (transitional morphology approaching adult features), and Stage V (complete sexual maturation). Investigators first explained the five developmental stages to the children through pictures and text, after which the children conducted self-assessments and reported the results to the investigators. Then, investigators classified boys’ pubertal status based on their reported genital development and age at first ejaculation; girls’ pubertal status was evaluated according to their reported breast development and age at menarche [28].

Parental education level was categorized into two groups: junior high school or below and high school or above. Family economic status was divided into three tiers: those with an annual income of ≤49k, 49–99k, and≥100k. This information was collected via the family information questionnaire completed by parents.

### 2.5. Statistical Analysis

Data cleaning and analyses were performed using SAS 9.4. In this study, 58,101 valid questionnaires were collected. Children with missing data on body composition, physical activity duration, or family information were excluded. Additionally, data were excluded if children had missing values for total energy expenditure or consumption or if they reported no consumption of grains, potatoes, or vegetables. Further exclusions included data where grain, potato, or lean meat consumption was below the 1st percentile or above the 99th percentile for their respective age groups. Ultimately, 48,305 children aged 6–17 years were included in the analysis. Yogurt consumption and body fat percentage (BFP) among children in different groups were described using medians (lower quartile and upper quartile), expressed as M (P25, P75).

Subsequently, quantile regression (PROC QUANTREG) was employed to analyze the relationship between children’s yogurt consumption and BFP. This method provides a more comprehensive view of the association than ordinary least squares regression, as it can reveal how the explanatory variable (yogurt consumption) differentially affects different quantiles of the outcome variable (BFP)—such as low, medium, and high body fat levels. Specifically, regression coefficients (β) and 95% confidence intervals were calculated to examine the correlation between increased yogurt consumption and changes in BFP across its distribution. A sex-stratified analysis was also performed to explore potential sex differences in the association between yogurt consumption and BFP. A two-tailed *p*-value < 0.05 was considered statistically significant.

## 3. Results

### 3.1. Sociodemographic Characteristics of Participants

A total of 48,305 children aged 6–17 years were included in this study, including 24,153 (50%) boys and 24,152 (50%) girls. Among them, children aged 6–10 years accounted for 39.6% (19,105), those aged 11–14 years made up 34.7% (16,785), and those aged 15–17 years constituted 25.7% (12,415). The sample sizes for boys and girls in each region of China are listed in Table 1.

### 3.2. Yogurt Consumption Status of the Study Subjects

For boys, the median daily consumption of yogurt was 28.6 g, 28.6 g and 21.4 g per day for the age groups of 6 to 10 years, 11 to 14 years, and 15 to 17 years, respectively (*p* < 0.05). For girls, they were 28.6 g, 29.6 g and 28.6 g for these age groups. (*p* < 0.05). For boys, the median body fat percentage was 19.6, 19.5 and 17.5 for the age groups of 6 to 10 years, 11 to 14 years, and 15 to 17 years, respectively (*p* < 0.05). For girls, they were 20.3, 26.4 and 31.0 for these age groups. (*p* < 0.05). Please refer to Table 2 for details.

### 3.3. Analysis of Between-Group Differences in Yogurt Consumption Levels and Body Fat Percentage

**Figure 1:**
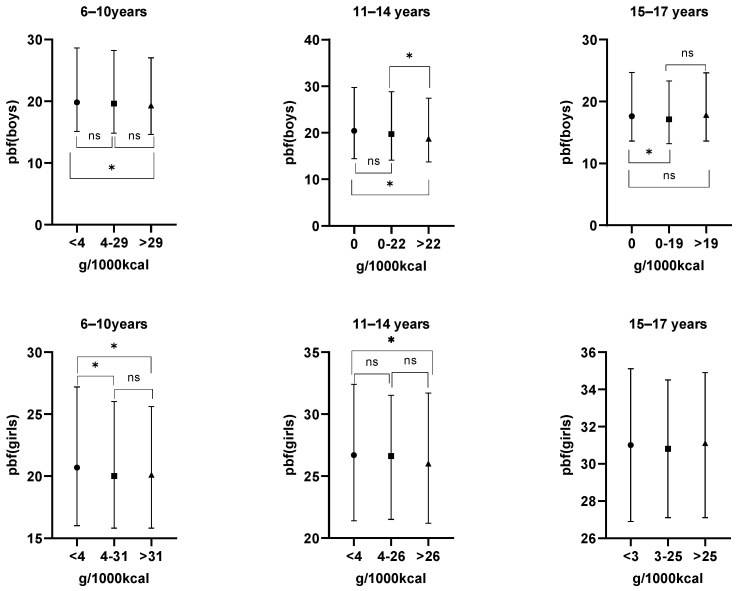
Comparison of body fat percentage of children aged 6–17 years across different yogurt consumption groups. Note: Differences in body fat percentage (BFP) across yogurt consumption groups were assessed using the Kruskal–Wallis test. In the figure, dots, squares, and triangles indicate the median yogurt consumption for each age group, while horizontal line segments depict the interquartile ranges (IQR) of BFP. An asterisk (*) denotes statistically significant differences in BFP among children with varying yogurt consumption levels, whereas “ns” indicates no statistical significance.

A significant inverse association was observed between yogurt consumption and body fat percentage across all age groups of boys in unadjusted analyses. The lowest body fat percentages were found at specific consumption levels: >29 g/day for ages 6–10 years (19.3), >22 g/day for ages 11–14 years (18.7), and 0–19 g/day for ages 15–17 years (17.1). All of these values were significantly lower than those in their respective groups with lower consumption (*p* < 0.05). For girls aged 6–10 years, the body fat percentage was 20.7 for girls consuming yogurt at <4 g/d, statistically higher than that of girls consuming yogurt at 4–31 g/d (20.0) and >31 g/d (20.1) (*p* < 0.05). For girls aged 11–14 years, the body fat percentage was 26.0% for girls consuming yogurt at >26 g/d, statistically lower than that of girls consuming yogurt at <4 g/d (26.7) (*p* < 0.05). Please refer to Figure 1 for details.

### 3.4. Association Between Yogurt Consumption and Body Fat Percentage: A Quantile Regression Analysis in Children (6–17 Years)

**Figure 2:**
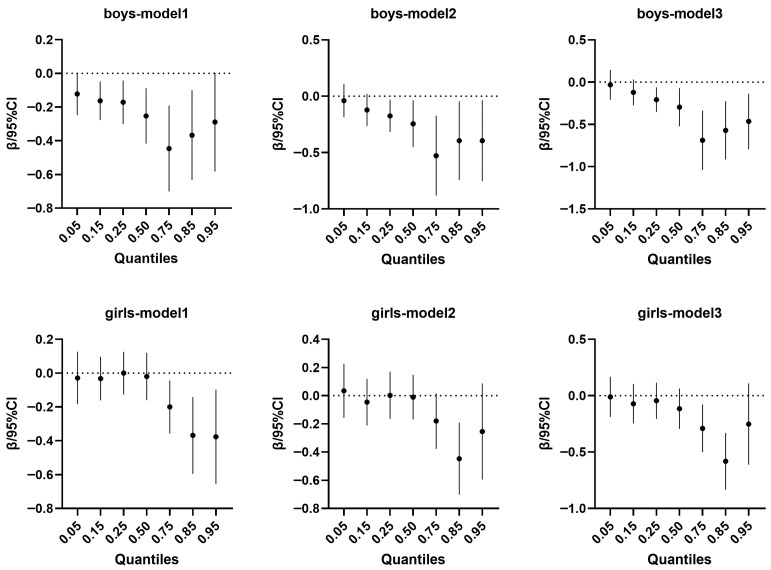
Quantile regression analysis of body fat percentage and yogurt consumption in children aged 6–17 years. Note: This diagram displays the quantile regression outcomes for the relationship between yogurt consumption and body fat percentage among children aged 6–17 years. The first model accounted for age and geographical distribution; the second model further incorporated pubertal stage, total daily caloric consumption, physical activity duration, sedentary behavior, annual family income, and parental education attainment; and the third model additionally included adjustments for other dietary consumptions. The black circles in the plot correspond to regression coefficients, with horizontal bars showing the associated 95% confidence intervals. A dashed reference line at zero denotes the null effect; estimates whose confidence intervals intersect this line are not statistically significant.

As illustrated in Figure 2. The quantile regression results indicated significant negative associations between yogurt consumption and body fat percentage (BFP) among boys across multiple quantiles. In Model 1 (adjusted for age and geographical distribution), significant correlations (*p* < 0.05) were observed at the 0.15, 0.25, 0.50, 0.75, and 0.85 quantiles. In Model 2 (further adjusted for pubertal stage, total daily caloric consumption, physical activity duration, sedentary behavior, annual family income, and parental education attainment), associations remained significant at the 0.25, 0.50, 0.75, 0.85, and 0.95 quantiles. Model 3, which additionally included adjustment for other dietary consumptions, showed consistent significance across the same quantiles as Model 2, with regression coefficients of −0.207, −0.300, −0.688, −0.570, and −0.465, respectively.

For girls, significant inverse associations between yogurt consumption and BFP were found predominantly at higher body fat quantiles. In Model 1, correlations were significant at the 0.75, 0.85, and 0.95 quantiles. Model 2 showed significance only at the 0.85 quantile, while Model 3, with full adjustment for dietary and lifestyle factors, revealed significant associations at the 0.75 and 0.85 quantiles, with corresponding regression coefficients of −0.290 and −0.582.

## 4. Discussion

In this cross-sectional study on yogurt consumption and body fat percentage in children, it was found that yogurt consumption was inversely proportional to body fat percentage among Chinese children. As we know, many studies exploring the effects of diet on body composition tend to employ multiple linear or logistic regression analysis [29]. Traditional linear regression primarily reveals trends within a dataset but fails to capture the data’s complete landscape. Moreover, as this method is rooted in the least squares principle, it exhibits high sensitivity to outliers [30]. Given that the effect of yogurt consumption on BFP may differ across varying body fat levels, quantile regression was adopted in this study. Unlike linear regression, this approach allows for analyzing relationships between variables across different quantiles, thus presenting a more comprehensive view of data distribution [31]. By minimizing weighted absolute errors, this method is less prone to the influence of outliers. As a result, it generates more robust estimates and precisely mirrors the actual relationships within the data.

This study found that yogurt consumption was negatively correlated with body fat percentage in children and adolescents. This conclusion is consistent with the results of a number of other observational studies and dietary intervention studies, which have shown that yogurt consumption exerts beneficial effects on body composition or has an inverse association with body composition [32,33,34,35]. For instance, a study conducted on 3786 American children aged 8–18 years [32] revealed that, compared with non-yogurt consumers, children who consumed yogurt showed significant improvements in several key anthropometric indicators: a 2.5 cm reduction in waist circumference, a 0.7 kg/m^2^ decrease in body mass index (BMI), and a 1.6 cm reduction in subscapular skinfold thickness (all *p* < 0.05). Unlike the cross-sectional design of the first study, the cohort study conducted by Panahi et al. (2018) provided more temporally relevant evidence regarding the association between yogurt consumption and body composition through a six-year follow-up [34]. The study further revealed that after adjusting for multiple confounders such as diet quality and physical activity, male yogurt consumers had a significantly lower body fat percentage than non-consumers (5% lower, *p =* 0.03). Moreover, a significant “group × time” interaction was observed during the follow-up period (with long-term yogurt consumption leading to a further reduction, resulting in a 4% lower body fat percentage compared to non-consumers, *p =* 0.01), suggesting that sustained yogurt consumption may offer cumulative benefits for body fat control. It is noteworthy, however, that no similar significant association was found in females, indicating that the effect of yogurt on body composition may differ by sex. The observed discrepancy may be attributable to factors such as hormonal variations, sex-specific patterns of adipose tissue distribution, and dietary differences. These findings underscore the need for further research to elucidate gender-specific physiological mechanisms.

A notable gender disparity was also identified in this study: yogurt consumption was inversely correlated with various levels of body fat percentage in males, whereas in females, such an association was only observed with higher levels of body fat percentage. This divergence may originate from physiological differences between sexes. Elevated circulating androgen levels typically contribute to increased lean mass synthesis and elevated basal metabolic rate in males [36]. Under such metabolic conditions, components of yogurt, such as whey protein and calcium, may promote more efficient fat oxidation and energy expenditure. Conversely, under the influence of estrogens, females exhibit a greater propensity for subcutaneous fat accumulation, particularly in the gluteofemoral region (hips and thighs), reflecting a sex-specific pattern of adipose tissue distribution [37]. Consequently, this inherent tendency toward adipose deposition may attenuate the responsiveness to the potential effects of yogurt on body fat reduction in females compared with males.

Yogurt is a semi-solid fermented dairy product produced by the lactic acid-producing bacteria Lactobacillus bulgaricus and Streptococcus thermophilus. Compared to milk, yogurt contains more vitamin B2, vitamin B12, calcium, magnesium, potassium, and zinc [33,38]. Clinical trials have shown that calcium in yogurt can regulate body weight and metabolism by reducing lipogenesis, stimulating lipolysis, and lipid oxidation [19]. Casein in yogurt inhibits angiotensin-converting enzyme, which inhibits the production of angiotensin II, which inhibits adipogenesis in adipocytes, thereby inhibiting fat deposition. Moreover, yogurt lactose is converted by bacteria into large amounts of lactic acid, which may affect the gut microbiota, thereby contributing to weight and metabolic control [18,19]. From the perspective of epidemiological research, studies have found that people who consume yogurt regularly have healthier dietary patterns (with higher consumption of fruits, vegetables, whole grains, and dairy products) and may have other differences in lifestyle (e.g., yogurt consumers usually exhibit higher levels of physical activity). These factors may help explain the research finding that people who consume yogurt regularly tend to have a lower body fat percentage [39,40]. However, it is noteworthy that some commercially available yogurts contain added sugar, which may not only increase the risk of excessive energy consumption but also adversely affect metabolic health through mechanisms such as impairing insulin sensitivity and promoting lipogenesis—impacts independent of its caloric contribution. These potential negative metabolic effects could mask the beneficial role of yogurt’s natural components in fat metabolism [41]. Therefore, future research is recommended to further differentiate the effects of sugar-free yogurt versus yogurt with added sugar on body fat regulation through methods such as randomized controlled trials so as to more accurately evaluate the actual role of yogurt in weight management.

This study has two main strengths: First, its large, nationally representative sample supports the generalization of conclusions. Second, it uses a semi-quantitative food frequency questionnaire (FFQ) for dietary assessment, which better captures school-age children’s long-term yogurt consumption and enables reliable analysis of its association with body fat. These strengths make the findings valuable for childhood overweight/obesity prevention/management and inform relevant strategies. However, the study has limitations, notably its cross-sectional design, which only reflects the yogurt consumption–body fat association at one time point (not dynamic changes over time). This means causal relationships cannot be established, limiting the interpretation of the underlying mechanisms.

## 5. Conclusions

This cross-sectional study revealed a statistically significant inverse association between yogurt consumption and body fat percentage among Chinese children and adolescents, with this relationship being particularly pronounced in subgroups with higher body fat percentage and in male individuals. It should be noted that body fat percentage, as a continuous variable, does not directly equate to a clinical diagnosis of overweight or obesity. The body fat percentage values presented in this study cannot precisely determine the “pathological” nature of adiposity. However, at the population level, elevated body fat percentage is highly consistent with increased risk of overweight and obesity. Therefore, even without establishing clear-cut-off points for “excess adiposity,” the observed inverse association between yogurt consumption and body fat percentage still provides important clues for public health: yogurt consumption may serve as a potential dietary factor in regulating overall energy balance and fat accumulation in children.

The findings of this study provide preliminary observational evidence for the potential role of yogurt in weight management. However, due to the inherent limitations of the cross-sectional design, causal relationships cannot be established. Future research should be advanced in the following directions: First, longitudinal designs or randomized controlled trials are needed to validate the long-term effects of yogurt consumption on body fat regulation and to determine causal direction. Second, gold-standard measurement methods, such as waist circumference, skinfold thickness, and dual-energy X-ray absorptiometry (DEXA), should be incorporated to definitively define clinical cut-offs for “excess adiposity,” thereby validating the specific clinical value of yogurt consumption for preventing or reducing pathological fat accumulation. Third, further differentiation between unsweetened yogurt and yogurt with added sugar is necessary to accurately evaluate their differential impacts on body fat regulation. These research directions will help inform the development of precise nutritional strategies based on gender and baseline adiposity levels and open up more diversified pathways for childhood obesity prevention and control.

## Figures and Tables

**Table 1:** Sociodemographic characteristics of participants.

	Total (N)	Boys (N, %)	Girls (N, %)
**Gender**	48,305	24,153 (50.0)	24,152 (50.0)
**Age** **(years)**			
6–10	19,105	9636 (39.9)	9469 (39.2)
11–14	16,785	8347 (34.6)	8438 (34.9)
15–17	12,415	6170 (25.5)	6245 (25.9)
**Area**			
Urban	24,896	12,410 (51.4)	12,486 (51.7)
Rural	23,409	11,743 (48.6)	11,666 (48.3)
**Region**			
North China	8280	4164 (17.2)	4116 (17.0)
Northeast China	8473	4283 (17.7)	4190 (17.4)
East China	7810	3874 (16.0)	3936 (16.3)
Central China	6419	3184 (13.2)	3235 (13.4)
South China	4828	2405 (10.0)	2423 (10.0)
Southwest China	7016	3518 (14.6)	3498 (14.5)
Northwest China	5479	2725 (11.3)	2754 (11.4)
**Pubertal development level**			
Tanner Stage I	17,173	10,033 (48.5)	7140 (34.2)
Tanner Stage II	6385	2596 (12.5)	3789 (18.1)
Tanner Stage III	8811	3737 (18.1)	5074 (24.3)
Tanner Stage IV	7538	3489 (16.9)	4049 (19.4)
Tanner Stage V	1676	844 (4.1)	832 (4.0)
**Medium- to high-intensity physical activity time (h/w)**	-	1.67 (0.5, 4.0)	1.42 (0.4, 3.3)
**Static behavior time (h/w)**	-	30.00 (24.7, 40.7)	32.25 (24.8, 41.1)
**Total energy consumption (kcal/d)**	-	2085.29 (1485.67, 3101.07)	1947.13 (1461.00, 2801.53)
**Father’s education level**			
Junior high school and below	23,115	11,458 (49.7)	11,657 (50.5)
High school and above	23,068	11,617 (50.3)	11,451 (49.6)
**Mother’s education level**			
Junior high school and below	23,853	11,788 (51.3)	12,065 (52.3)
High school and above	22,203	11,180 (48.7)	11,023 (47.7)
**Total household income last year**			
≤49 k	11,440	5557 (32.1)	5883 (34.1)
49~99 k	11,030	5523 (31.9)	5507 (31.9)
≥100 k	12,097	6225 (36.0)	5872 (34.0)

Note: Children’s gender, administrative region, pubertal development status, parental education level, and total household income in the past year were presented as N (%). Duration of physical activity, sedentary time, and total daily energy consumption were continuous variables and are expressed as medians with interquartile ranges.

**Table 2:** Daily consumption of dairy yogurt for children aged 6–17 years.

	BFP				Yogurt Consumption					
Sex	Age (Year)	M (*P*_25_, *P*_75_)	χ^2^	*p*	M (*P*_25_, *P*_75_) g (mL)	χ^2^	*p*	M (*P*_25_, *P*_75_) g (mL)/1000 kcal	χ^2^	*p*
boys	6~10	19.6 (14.8, 27.85)	228.325	<0.001	28.6 (0.0, 79.5)	23.619	<0.001	16.2 (0.0, 39.8)	236.217	<0.001
	11~14	19.5 (14, 28.6)			28.6 (0.0, 85.7)			10.2 (0.0, 31.3)		
	15~17	17.5 (13.5, 24.3)			21.4 (0.0, 85.7)			8.0 (0.0, 27.9)		
	Total	19 (14.1, 27.1)			28.6 (0.0, 82.2)			11.7 (0.0, 33.9)		
girls	6~10	20.3 (15.8, 26.2)	5568.843	<0.001	28.6 (0.0, 85.7)	12.323	0.002	17.0 (0.0, 40.8)	31.407	<0.001
	11~14	26.4 (21.3, 31.9)			29.6 (0.0, 85.7) *			14.1 (0.0, 35.9) *		
	15~17	31 (27, 34.9)			28.6 (0.0, 85.7) *			13.5 (0, 34.7) *		
	Total	25.7 (19.6, 31.8)			28.6 (0.0, 85.7) *			15.0 (0.0, 37.5) *		

Note: In this study, the Kruskal–Wallis test was used to analyze differences in yogurt consumption among children of different age groups, while the Wilcoxon rank-sum test was employed to compare yogurt consumption between boys and girls within the same age group; * indicates that the difference in yogurt consumption between boys and girls within the same age group was statistically significant.

## Data Availability

The data are not publicly available due to privacy/ethical restrictions.

## Abbreviations

The following abbreviations are used in this manuscript:

BFP	body fat percentage
BMI	body mass index
